# 

**Published:** 1882-02

**Authors:** 


A MONTHLY JOURNAL OF MEDICAL SCIENCE.

*"Magna est veritas, et prævalebit."*

E. J. LEE, M. D.

EDITOR.

CONTENTS:

(See inside of Cover.)

CONTRIBUTORS:

Drs. H. C. Allen, T. F. Allen, Edward Bayard, J. B. Bell, E. W. Berridge, Geo. H. Clark, A. C. Cowperthwaite, Ad. Fellger, Wm. J. Guernsey, W. A. Hawley, John Hall, W. M. James, W. H. Jenny, Ad. Lippe, C. Lippe, Malcolm MacFarlan, Thos. Moore, C. F. Nichols, C. Pearson, T. F. Pomeroy, Leila A. RenDell, C. Carleton Smith, P. P. Wells, Wm. P. Wesselhoeft, David Wilson, (London), T. P. Wilson, and many others.

NOTICE.

***All articles for publication, books for review, business communications, etc., must be sent to Editor, 2109 Chestnut Street, Philadelphia.***

PHILADELPHIA:

No. 2109 CHESTNUT STREET.

LONDON.

ALFRED HEATH & CO., SOLE AGENTS FOR GREAT BRITAIN,

114 Ebury Street, Eaton Square.

***Yearly Subscription, $2.00, invariably in advance.***

Entered at the Philadelphia Post-Office, as Second-Class Mail Matter.

